# Phlegmasia Dolens, or What?1Read before the Iowa-Nebraska Veterinary Medical Association, Omaha, Oct., 1901.

**Published:** 1902-07

**Authors:** C. McKim

**Affiliations:** Norfolk, Neb.


### PHLEGMASIA DOLENS, OR WHAT?[1]

By C. McKim, M.D.C.,
NORFOLK, NEB.

In selecting this subject for my paper before this Association I have not done so thinking I know much about it, or that I can give you anything new or of importance in regard to it; but its being of rare occurrence and having happened in my practice at two different times, once in a cow and once in a mare, I was led or compelled to give it some extra study, so thought it might be of interest to you.

The definition of the word phlego is to inflame, and of phlegmasia dolens, or phlegmasia alba dolens, an acute œdema of the leg from venous obstruction, a white, firm swelling of the legs after delivery.

On March 25, 1898, I was called to see a cow belonging to our dairyman, which he said was one of his best milkers. On my arrival at his farm I found the patient in his cow stable (which was a very good one, well built, ventilated, drained and well lighted); the owner informed me the cow had calved a few days before—not stating just how many days—and apparently had no trouble in giving birth to the calf. The owner was also sure she had passed all of the placenta or after-birth, and he had thought, up to the day before my visit, she had been doing very well. Then he noticed a slight discharge passing from the vulva; she was restless and refused her feed, or only ate indifferently. She also strained some, and made frequent attempts to micturate. By examination per vulva and vagina, I found the mucous membrane injected; the os uteri was so firmly constricted I could not at first pass my index finger into it. I removed from the vagina long strings or masses of dark yellow or chocolate-colored mucus. The pulse was quick and frequent; I have forgotten now the degree of fever, but her temperature was elevated. She would lie down and be disinclined to rise, lying on her side and grunting.

My diagnosis was acute septic metritis.

As the cow had received good care and feeding and been in warm quarters, I could not say it was caused by neglect or taking cold, but considered it due to absorption of some septic

[1] Read before the Iowa-Nebraska Veterinary Medical Association, Omaha, Oct., 1901.

material, probably part of the placental membranes; but I could not dilate the os sufficiently to get my hand into the uterus to make an examination. I introduced my female catheter through the os and flushed out the uterus and vagina with a solution of permanganate of potash, having the water as hot as I could bear my hand in it. This I injected with my injection-pump.

This was repeated every other day at my visits, as the owner could not attend to it himself. It should have been done once or twice a day. After using the permanganate a few days I alternated with a lysol solution.

I also prescribed a fever mixture, and later a tonic or stimulant.

With this treatment the cow improved slowly, but on April 1st the owner came in and told me the cow was lame in the left hind leg. I promised to go out the next day. I did so, and found her suffering from a good deal of pain in the leg; it was swollen at and a little above the fetlock, and was hot and painful to the touch. I asked the owner if she could have hurt herself in any way, but he could not account for it at all. There were no signs of any injury. I, however, prescribed for a sprain—hot fomentations and bathing with a liniment, and bandaging. At my next visit, imagine my surprise and puzzle in finding the cow's leg swollen nearly up to the body, the swelling being œdematous, the lymphatics on the inside of the leg enlarged and corded. My first impression was: "Is it a case of lymphangitis?" But I thought not. I continued my hot applications and liniment, also the fever mixture, and instructed the owner to come in and let me know how it was the following morning.

I admit I was bothered to account for the conditions presenting in the case, unless I might call it septic infection from the uterus, and I did think of a pelvic abscess, but could not locate one, and the limb swelling first at the fetlock was a puzzle; but now I can account for it, if there was a venous obstruction. I had, a short time before this, secured Dalrymple's *Veterinary Obstetrics.*

I will now give you his description of my case, which I concluded was phlegmasia dolens:

"This condition," he says, "is more common in the human subject than in the lower animals. It appears, generally, a few days after parturition. It is due to an obstruction of the lymphatics of one or more limbs, or of the femoral or femoral and iliac veins, and is followed by œdema of the affected limbs. The cause is believed to be pressure of the uterus or thrombus, due to obstruction by some foreign material from the uterus."

The *American Text-book of Obstetrics* calls it phlegmasia alba dolens, and says: "It may be due either to phlebitis or to cellulitis. Often both conditions are combined. In the phlebitic form one or more veins form solid strings, and below the obstruction the extremity becomes œdematous and swollen. In the cellulitic form the skin is white or pink, tense and hard, one or both legs swell, and the epidermis may be lifted by a serous fluid, forming large vesicles. The inguinal glands swell. Suppuration and mortification may spread destruction in the connective tissue under the skin or between the muscles. This pernicious form, however, is rare."

(But in my case in the mare, as I will describe, I had all the above conditions.)

"Commonly the inflammation begins the second week after confinement. The lymphatics on the inside of one or both hind limbs become enlarged and corded, somewhat resembling lymphangitis. This is followed by œdema of the whole limb; the animal loses the power of the limb (in my cases this was only partial), first *at the fetlock*, then all of the joints become affected, and ultimately the patient gets down. On examination the limb is found to be swollen, hot, and very painful. The disturbance is considerable, and much fever is present; the pulse is hard, quick, and frequent; there is inappetence, and the secretion of milk much diminished. If the patient is carefully treated these symptoms gradually disappear, but in some cases it runs on. Inflammation of the tissues of the limb generally takes place, and abcesses form, chiefly at the hock. The animal may die from anæmia, septicæmia, or traumatic fever" (Dalrymple).

All these symptoms were marked in my case: the cow gave hardly any milk; a large abscess formed at the external lateral aspect of the hock, and I could pass my long probe for twelve inches up under the perforans and perforatus tendons and up under the postero-internal aspect of the limb. The discharge was of a yellow or creamy color, and the owner asked me if it was not the milk coming down that way.

Dalrymple says: "If the patient be a mare put her in slings. Give a laxative in all animals, followed by febrifuges and diuretics. Where the limb is very painful, hot fomentations may be used in the first stage, and judicious scarification if the effusion is considerable. Patients that will not permit of slinging should be turned every three or four hours. When the acute stage has been passed, bandaging will assist absorption, and diuretics, absorbents, and tonics should be given.

"The phlegmasia usually runs its course in from three to six weeks, and ends in resolution; it may pass into suppuration, and the patient still recover. As a rule, the thrombus is absorbed and the swelling subsides."

I followed the above treatment as suggested. The abscesses I treated twice daily with hot poultices and syringing out the cavities, when poultices were changed, with a lysol solution. The discharge per vagina was stopped, and the animal made a recovery in about six weeks.

On April 13, 1900, I was called out to see a large black mare. She had a colt about ten days old; the owner had thought her all right until this time, but she now began to refuse her feed, and seemed to be in pain. There was a discharge from the vagina, which he said had been so since she foaled. I could discover nothing but the discharge, and she disliked to have me examine the vagina, as if it gave her pain.

I left a wash for the vagina and a tonic and fever mixture—her temperature was about 102° F. The owner reported in a day or so that she was doing well.

On April 23d he came for me again, and when I got out to his place I found the mare in a good deal of pain; the left fetlock swollen and hot; she could not bear her weight on it very well. I could find no swelling or soreness higher up at this visit, but I suspected it was phlegmasia, and prescribed as above. On the 28th her leg was swollen nearly to the body, and all other symptoms of the disease were present. In a few days a hard, firm swelling appeared over the gluteal muscles of the left side, and I could detect it with my hand in the vagina. On May 5th, when I went out, I found her down and unable to rise. The owner said we might as well kill her. I found the abscess in the gluteal muscles ready to open, and made a long free incision, and I believe more than a gallon of pus escaped. This should have been opened several days earlier, but the owner had been in, and told me not to come out, as he thought it no use, but I went out on this trip anyway. After the pus escaped I took my long seton needle and passed it into the cavity until I could feel it through the vagina, and I made a good free opening into the vagina and about eight inches in from the vulva. Then I flushed the cavity out thoroughly with a permanganate wash. The mare got up before I left. I treated her similar to the cow, and she made a nice recovery in six or eight weeks, and is still alive and working, although away up in her teens.

---

A New Tryponosoma in Cattle. Theiler, of Pretoria, has found in cattle a new tryponosoma, which Laveran has called tryponosoma Theileri. This parasite, which is peculiar to bovida and presents special characteristics, is pathogenic. It produces anæmia with or without fever. After the fever it may be found in the blood during several weeks. More rarely there is observed a pernicious anæmia with rapid destruction of the red blood cells and rapidly terminating in death. At the post-mortem, enlargement of the spleen and subpericardial ecchymoses are found; the red corpuscles are diminished in number, of variable dimensions, and some are nucleated.

In those cases of pernicious anæmia the tryponosoma cannot be found in the blood, but this blood inoculated into two calves reproduced the disease with the tryponosoma found in the blood.—*Rec. de Med. Vet.*

Parasites in the Blood of Epileptic Persons. Essential or idiopathic epilepsy is regarded as a neurosis with anatomical lesions not well understood. Bra has found a micro-organism in the blood abstracted from the veins of the forearm of seventy epileptics.

During the intervals between the paroxysm the examination of the blood is negative, but before, during, and immediately after an attack the parasite can be seen under a magnifying power of at least 500. This micro-organism consists of small points of at least 1 $\mu$, feebly refracting, isolated, or in chains of six or eight, and endowed with very rapid movements. The chains, usually formed by larger bodies at each end, are endowed with undulatory movements. When the chains are absent this organism appears as cocci-like bodies, isolated, free, attached to a red corpuscle or surrounded by a phagocyte

This microbe will stain, although not very well, with eosin, phenicated thionin and Kühne's blue. It is anaërobic and will grow on bouillon, gelatin and potato.—*Comp. rend. de l'Acad. de Science de Paris.*

